# Driving a Superconductor to Insulator Transition with Random Gauge Fields

**DOI:** 10.1038/srep38166

**Published:** 2016-11-30

**Authors:** H. Q. Nguyen, S. M. Hollen, J. Shainline, J. M. Xu, J. M. Valles

**Affiliations:** 1 Department of Physics, Brown University, Providence, RI 02912 USA; 2 Nano and Energy Center, Hanoi University of Science, Vietnam National University, Hanoi, Vietnam; 3 Department of Physics, University of New Hampshire, Durham, NH 03824 USA; 4 National Institute of Standards and Technology, 325 Broadway, Boulder, Colorado, 80305, USA; 5 School of Engineering, Brown University, Providence, RI 02912, USA

## Abstract

Typically the disorder that alters the interference of particle waves to produce Anderson localization is potential scattering from randomly placed impurities. Here we show that disorder in the form of random gauge fields that act directly on particle phases can also drive localization. We present evidence of a superfluid bose glass to insulator transition at a critical level of this gauge field disorder in a nano-patterned array of amorphous Bi islands. This transition shows signs of metallic transport near the critical point characterized by a resistance 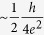, indicative of a quantum phase transition. The critical disorder depends on interisland coupling in agreement with recent Quantum Monte Carlo simulations. We discuss how this disorder tuned SIT differs from the common frustration tuned SIT that also occurs in magnetic fields. Its discovery enables new high fidelity comparisons between theoretical and experimental studies of disorder effects on quantum critical systems.

A random gauge field adds random increments to the phase of a particle as it traverses a system. It appears as a random phase factor in the site to site tunneling integral in tight binding models. For the most familiar random gauge field, a random magnetic field with zero mean, the phase shifts take the form 
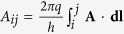
 for a charge q moving from *i* to *j* in a magnetic vector potential, **A**. The effects of random gauge fields, also called gauge field disorder, have been considered in attempts to describe anomalous transport in the normal state of high temperature superconductors^1^, graphene^2^,^3^, the *ν* = 1/2 state in two dimensional electron gases^4^,^5^, and photons in solid state structures^6^.

Fluctuations in gauge fields influence fermions and bosons distinctly. Magneto-transport experiments on rippled graphene suggest that they counteract Anderson localization of fermions^2^,^3^,^7^. Similarly, models show that random Chern-Simons gauge fields produce the nearly metallic rather than localized transport associated with the *ν* = 1/2 state^4^,^8^. On the other hand, gauge field fluctuations appear to destroy superfluidity and tend to localize bosons^1^. Attempts to explain the normal state transport of high *T*_*c*_ superconductors using resonating valence bond models have led investigators to consider how random gauge fields affect bosons in two dimensions^1^. Fluctuations in the gauge field appear to suppress Bose condensation and thus, superfluidity at finite temperatures in those treatments of t-J models^1^.

Multiple groups have manipulated and engineered gauge fields to address new physics^5^,^9^,^10^,^11^. A few have applied spatially random magnetic fields to two dimensional electron systems^5^,^10^,^11^ to investigate models of the *ν* = 1/2 fractional quantum hall state. The motivation to create ever more versatile quantum simulators of many body systems has led to methods for producing artificial gauge fields in uncharged systems, such as cold neutral atom or quantum optics^6^,^12^,^13^. Particularly germane to the current report, a couple of groups created disordered gauge fields in Josephson Junction Arrays (JJA). They fabricated arrays with positional disorder to produce a random amount of flux per plaquette in the presence of a transverse field^14^,^15^. Their studies focused on the effects of this disorder on the classical Berezinski-Kosterlitz-Thouless transition^16^,^17^. Here, we employ a similar approach to investigate the effects of random gauge fields on the quantum superconductor to insulator transition. We show that strengthening a random gauge field weakens a superfluid state and can even drive a low superfluid density superconductor into an insulating phase.

These investigations employ films patterned into arrays that are on the superconducting side of a thickness tuned superconductor to insulator transition (Fig. 1b)^18^. It is helpful to consider their behavior in the light of the quantum rotor model that is commonly used to describe the SIT^19^,^20^,^21^,^22^. Its Hamiltonian is given by:


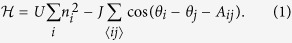


*n*_*i*_, the number operator for Cooper pairs and *θ*_*j*_, the phase operator on node *j* satisfy [*n*_*i*_, *θ*_*j*_] = i*δ*_*ij*_ (see Fig. 1c). The first term is an onsite Coulomb energy of strength *U* that tends to localize Cooper pairs to individual nodes. The second term, which sums over nearest neighbors, competes with the first by promoting phase coherence and a delocalized superfluid state. The internode coupling *J* is proportional to the amplitude of the superconducting order parameter on the nodes and tunneling coupling between nodes. The argument of the cosine is the gauge invariant phase shift, *η*_*ij*_ = *θ*_*i*_−*θ*_*j*_−*A*_*ij*_, for a boson tunneling directly from island *i* to island *j*. In zero magnetic field, *H* = 0, and for perfectly ordered arrays, this model exhibits a superconductor to insulator transition at a critical coupling *K*_*c*_(0) = (*J*/*U*)_*c*_ = 0.206^21^,^23^ below which quantum phase fluctuations drive Cooper pair localization. Now consider commensurate magnetic fields for which Σ*A*_*ij*_ = 2*πn* around a plaquette or the number of flux quanta per plaquette *ϕ*/*ϕ*_0_ is an integer, *n*^20^,^21^. *ϕ*_0_ = *h*/2*e* is the superconducting flux quantum. This model predicts that *K*_*c*_(*n*) = *K*_*c*_(0) provided *J* and *U* do not depend on magnetic field.

In a geometrically disordered array, the critical coupling, *K*_*c*_, grows with commensurate magnetic field strength according to simulations of the quantum rotor model^21^,^24^. To see how this effect occurs, consider the amplitude for a Cooper pair tunneling from site *i* to *j*. The associated tunneling probability amplitude is given by the superposition of all paths connecting these sites. These paths interfere constructively to give the greatest net amplitude when *η*_*ij*_ = 2*πn*, for integer *n*, along every link in the array. This condition holds for ideal ordered arrays at commensurate fields. In arrays with a distribution of unit cell areas (like Fig. 1d), however, it is only possible to approximate commensurability by making 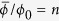 for the average flux per plaquette. At this average condition, the *A*_*ij*_ vary randomly with a mean of 0 (as in Fig. 1e–h). The random phase shifts induced by this gauge field weaken the constructive interference effect described above. The associated reduction in the tunneling probability amplitude makes the system more susceptible to phase fluctuations. *K*_*c*_ increases to compensate. This dependence of *K*_*c*_ on random gauge field strength makes it possible to use a series of commensurate fields to tune through a SIT. As we describe below, this Random Gauge Field Tuned SIT joins the general class of disorder tuned quantum phase transitions^19^ as an example that is particularly amenable to theoretical analysis.

## Results and Discussion

**Figure 1:**
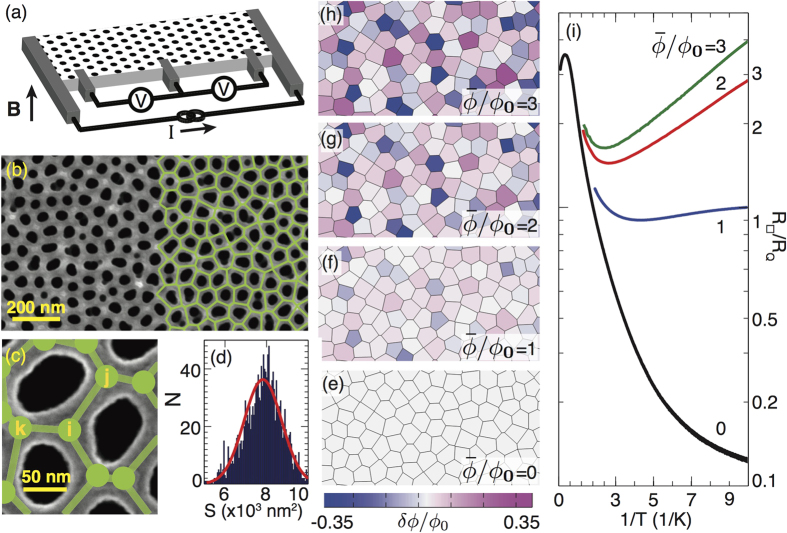
Tuning Random Gauge Fields. (**a**) Schematic sample measurement setup. A uniform magnetic field **B** is applied perpendicular to the sample plane. (**b**) Scanning electron microscope image of an amorphous Bi nano-honeycomb film. The overlaid green network of links defining individual array cells was obtained using a triangulation method. (**c**) Magnified region of (**b**) showing dots to denote nodes. (**d**) Distribution of cell areas defined by the links between nodes with its Gaussian fit (red line: 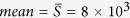 nm^2^ and *σ* = Δ*S* =0.92 × 10^3^ nm^2^). (**e**–**h**) Maps of the deviation of the magnetic flux through a cell from the average value, *δϕ*, in units of the flux quantum, *ϕ*_0_, for commensurate fields 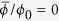, 1, 2 and 3. The random variations in *δϕ* imply random variations in the line integral of the gauge field *A*_*ij*_ along links that grow proportionally with 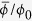. (**i**) Sheet resistance as a function of inverse temperature at commensurate magnetic fields that are well below the estimated upper critical magnetic field for this *R*_*N*_ = 20 kΩ film. The *R(T*) at low temperatures evolve from superconducting to insulating characteristics with increasing 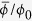 (see text).

We produced random gauge fields by applying commensurate magnetic fields to films patterned into a geometrically disordered hexagonal array (Fig. 1b). We thermally evaporated Sb and then Bi onto cryogenically cooled anodized aluminum oxide substrates with surfaces perforated by a disordered triangular array of holes^25^. Similarly produced nano-honeycomb (NHC) films undergo a localized Cooper pair to superfluid transition with increasing deposition^18^. The nodes (Fig. 1c), which have a relatively larger thickness than the links due to undulations in the substrate surface, harbor more Cooper pairs compared to the links connecting them^26^. The geometric disorder of the NHC array is apparent in the distribution of unit cell areas obtained by reconstructing the array with a triangulation algorithm (Fig. 1d)^25^.

We employed our most strongly geometrically disordered arrays for these experiments. This choice enabled us to apply strong gauge field disorder at fields, 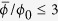, that were well below the upper critical magnetic field, 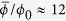
27. In this low field regime the magnetoresistance exhibits a decaying oscillation pattern with minima at the commensurate fields^25^ (see Supplemental Information). Within the quantum rotor picture, the oscillations result from the modulation of the *cosine* term, which leads to a modulation of the average Josephson coupling in the array^23^. The decrease in the oscillation amplitude can be attributed quantitatively to the growth of flux disorder with increasing field. Previous experiments^14^ and simulations^28^ on disordered square arrays and simulations of disordered hexagonal arrays^29^ show that oscillations disappear when 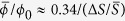. This relation implies a maximum of 3 oscillations for the NHC film shown in Fig. 1 for which 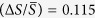, in good agreement with the data (see ref. 25 and Supplemental Information). This agreement supports discussing the ensuing phenomena in terms of the quantum rotor model with a distribution of plaquette areas and a field independent *J*. Other potentially confounding field effects on *J* due to pairbreaking^27^ or mesoscopic fluctuations^30^ were minimized by staying well below the upper critical magnetic field (see Supplementary Information).

We characterize the strength of the gauge field disorder by the variance in the distribution of *A*_*ij*_, Δ*A*_*ij*_, and an associated phase randomization length, *L*_*θ*_. Δ*A*_*ij*_ can be related to the variance in the flux per unit cell^31^ in the strong disorder limit where variations in *A*_*ij*_ and the flux per plaquette exert similar effects^16^. For an array with *n*_*L*_ links per plaquette and fractional variance in plaquette areas 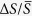:


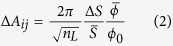


The maps in Fig. 1e–h show how this disorder grows from zero with increasing commensurate magnetic field. The phase randomization length gives the average distance that a Cooper pair travels before the gauge disorder has completely randomized its phase. To calculate it, consider particle trajectories consisting of *N* steps along links of average length *a*. At each step, there is a random phase shift of average size Δ*A*_*ij*_, so that the distribution of the sum of the phase shifts will have a width 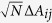. When that width becomes of order *π* the distribution of phases covers most of the unit circle. This maximal phase randomization occurs on length scales of order *L*_*θ*_ = *a(π*/Δ*A*_*ij*_)^2^ where *a* is the lattice constant. Thus, *L*_*θ*_ provides a phase coherence length over which Cooper pair constructive interference effects can promote delocalization. At the maximum field employed in these experiments, 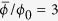, Δ*A*_*ij*_ = 0.88 radians and 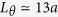 for *n*_*L*_ = 6 and 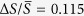. It is illuminating to note that *L*_*θ*_/*a* ≤ *n*_*L*_ at 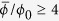 where no modulation effects are apparent in the magnetotransport.

**Figure 2:**
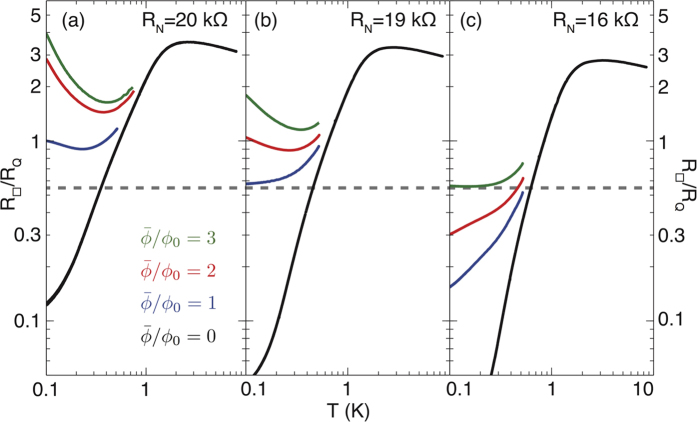
Coupling Dependence of the Random Gauge Field Tuned SIT. (**a**–**c**) *R*_□_(*T*) of films with three different *R*_*N*_ at 4 levels of gauge field disorder as reflected by the average flux per plaquette. The dashed line is an estimate of the critical resistance for the transition (see text). 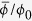 = 0, 1, 2, 3, corresponds to ∆*A*_*ij*_ = 0, 0.29, 0.59, 0.88, respectively.

Transport measurements in the low temperature limit indicate that Cooper pairs become more localized with increasing commensurate magnetic fields (Fig. 1i). At temperature, T = 100 mK, *R*_□_ rises monotonically by a factor of 15 (Fig. 2a inset). This rise spans the resistance quantum for pairs *R*_*Q*_ = *h*/(2*e*)^2^. *R*_*Q*_ normally separates conduction by delocalized charge 2*e* carriers in a metallic or superconducting state from the incoherent tunneling between localized states. This separation is evident in Fig. 1i as the coincident change in the temperature dependence of the resistance from *dR*_□_/*dT* < 0 for 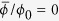 to *dR*_□_/*dT* > 0 for 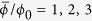. The *R*_□_(*T*) develop an exponential dependence consistent with thermally activated tunneling with an energy barrier that increases with 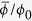. We reproduced this evolution of *R(T*) in a second sample on another substrate.

We attribute this dramatic transformation from superconducting to insulating behavior (cf. Fig. 1i) to the influence of gauge field disorder. Ordered arrays do not exhibit this behavior. According to experiment^32^ and the Hamiltonian in Eq. (1)^21^, a film that superconducts in zero magnetic field, superconducts at all commensurate fields. Moreover, this random gauge field tuned transition is distinct from the magnetic field tuned superconductor to insulator transitions (BSITs) that appear at incommensurate fields^25^,^33^. Incommensurate fields have net vorticity that frustrates phase ordering to make an array more susceptible to phase fluctuations^20^. This frustration drives the BSITs that have been observed in ordered, micro-fabricated JJAs^32^,^34^. Thus, BSITs are frustration driven and random gauge field tuned SITs are disorder driven.

The critical gauge field disorder for this SIT depends on the zero field coupling constant, *K* = *J*/*U*, which varies with the normal state sheet resistance. Figure 2a–c shows the *R*_□_(*T*) of three films with different *K* at commensurate fields. They are on the same NHC substrate (Fig. 1a) so that their random gauge fields have the same magnitudes Δ*A*_*ij*_ = (0, 0.29, 0.59, 0.88) for 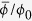 = (0, 1, 2, and 3), respectively. To estimate the coupling constants, we presume that *J*∝*T*_*c*_/*R*_*N*_ in accord with the scaling of the coupling energy of a Josephson tunnel junction and that the single island charging energy *U* is fixed by the geometry of the substrate. In Fig. 2, *K* increases from left to right as *R*_*N*_ decreases and *T*_*c*_ concomitantly increases. The *R*_*N*_ = 20 kΩ film shows a superconducting characteristic (i.e. *dR*_□_/*dT* > 0 as *T* → 0) only for 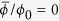. It is tuned to an insulating characteristic (i.e. *dR*_□_/*dT* < 0 as *T* → 0) for 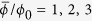. At 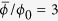 the transport fits an activated temperature dependence with an activation energy of 131 mK (see Fig. 1i). With increasing *K*, the transition from superconducting to insulating behavior moves to higher 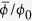 (Fig. 2b,c). In fact, the most disordered gauge field barely tuned the most strongly coupled 16 kΩ film into the insulating phase. Altogether, the greater the difference between *K* and *K*_*c*_(0), the larger Δ*A*_*ij*_ must be to drive the SIT.

**Figure 3:**
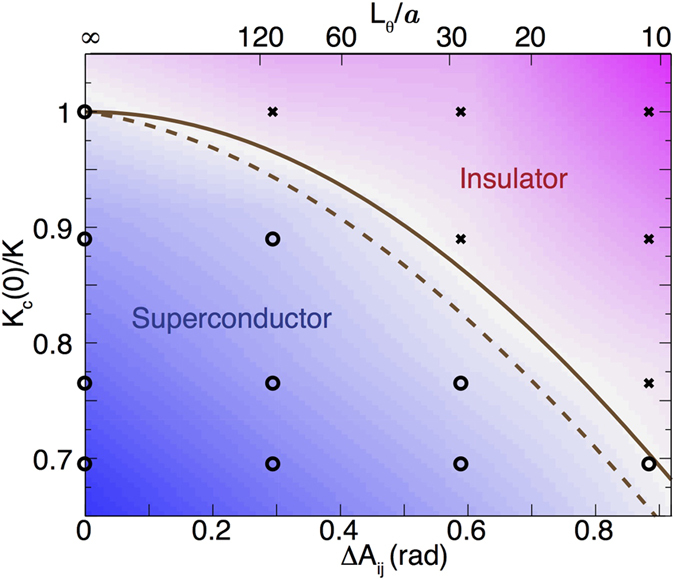
Phase Diagram of the Random Gauge Field Tuned SIT. The inverse coupling constant values correspond to the four films with [(*R*_*N*_(*k*Ω), *T*_*c*_(*K*)) = (20.3, 1.1), (19.2, 1.2), (17.5, 1.28), (16.7, 1.34)] where *Tc* is the temperature at which the resistance drops to 80% of its normal state value. They are normalized to the 20 *k*Ω film value. Open circles correspond to films with superconducting characteristics and crosses correspond to films with insulating characteristics. The shading qualitatively represents the low temperature *d*log (*R*_□_)/*dT*. The lines give predictions for the superconductor-insulator phase boundary derived from figure 18 of ref. 21 without (solid) and with (dashed) magnetic pair breaking taken into account (see text).

Condensing these observations into a phase diagram of inverse coupling constant, *K*_*c*_(0)/*K*, versus gauge disorder, Δ*A*_*ij*_ phase diagram (Fig. 3) enables comparison with simulations. Any phase boundary separating superconducting and insulating films must have a negative slope implying that the critical coupling for the transition increases with gauge field disorder. This behavior agrees well with predictions (solid line in Fig. 3) from Quantum Monte Carlo simulations of a (2 + 1)D XY Model Hamiltonian (Equation (1)) on a square array by Kim and Stroud^21^. Those simulations showed that the ground state transforms from a phase ordered, superconducting Bose glass to a Mott insulator at a critical coupling that decreases with gauge field disorder. More recent simulations of a hexagonal array using path integral based methods yielded a nearly identical phase boundary^29^. It is important to note that the simulations do not include any magnetic field dependence of *J*. However, we estimate (see Supplemental Information) that *J* decreases by the factor 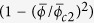 due to the finite size of the dots in the NHC films^26^. Multiplying the ideal array phase boundary line by this factor using an upper critical field of 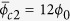, produces the dashed line, which deviates from the simulations by no more than 7% at the highest field. Altogether, this comparison reveals that the data are consistent with gauge field disorder driving a Bose glass to Mott insulator quantum phase transition.

Furthermore, there is evidence of a quantum critical resistance^35^ at this random gauge field tuned SIT. Typically, quantum critical resistances are obtained through magnetic field scaling analyses of R(T) measured at a number of closely spaced magnetic fields around the critical magnetic field. This procedure cannot be carried out reliably on the R(T) showing this random gauge field tuned transition because they are obtained at widely spaced magnetic fields that are set by commensurate field values. Nevertheless, an estimate can be made without the scaling analysis. The low temperature tails of the *R*_□_(*T*) in Fig. 2 sweep continuously from a positive to a negative slope with increasing 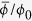 suggesting that *R*_□_(*T*) becomes temperature independent at a critical amount of disorder. Such a metallic flattening is most evident in the *R*_□_(*T*) for *R*_*N*_ = 19 kΩ (Fig. 2b), 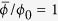 and *R*_*N*_ = 16 kΩ (Fig. 2c), 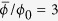, which appear to asymptote to 3.5 kΩ. While this asymptotic separatrix is consistent with the *R*_*N*_ = 20 kΩ data (Fig. 2a), none of those *R*_□_(*T*) become level at low *T*. We conjecture that the metallic behavior appears only at a specific coupling constant for each integer 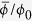. The data are consistent with a critical resistance *R*_*c*_ ≈ 0.5*R*_*Q*_ independent of coupling constant. The Quantum Monte Carlo simulations that apply most directly to the present experiment predict about a factor of 3 variation in *R*_*c*_^21^ that brackets the experimental value. The prediction that *R*_*c*_ varies with *K*, however, is inconsistent with the data.

## Conclusion

We introduced a method to impose a random gauge field on superconducting thin films near a thickness tuned SIT. We observed that the films can be tuned across the SIT by increasing the amplitude of the random gauge field in accord with numerical predictions^21^. Much about this random gauge field tuned transition remains to be explored including the response of the insulator to gauge field disorder and the discrepancy between theory and experiment on the variation of *R*_*c*_ in the quantum critical transport. The capability to tune coupling and disorder independently afforded by the NHC substrate platform will be useful for such studies. In general, experimental realizations of purely disorder driven localization transitions like the one presented here are difficult to achieve. Theoretically, they have been studied extensively^19^ using models with potential disorder that give rise to Anderson localization driven metal to insulator transitions and phase fluctuation dominated superconductor to insulator transitions, for example. The challenge for experiments has been creating systems in which the potential disorder can be tuned independently of electron electron interactions. The development of models like that of Kim and Stroud^21^ and others that employ geometrical or bond disorder^22^,^36^ and this study present new opportunities for isolating disorder’s influence on quantum phase transitions.

## Methods

The Anodized Aluminum Oxide substrates with disordered hole arrays were produced by covering a thin sheet of aluminum with teflon and anodizing them using standard methods^25^,^37^. The films were created by thermally evaporating a wetting layer of Sb and a series of Bi layers onto the substrates while they were held at 8 K inside a dilution cryostat. Sheet resistances, *R*_□_, were measured on a 1 mm^2^ area of film *in situ* using standard 4 point low frequency techniques (Fig. 1a)^18^. Perpendicular magnetic fields were applied with a superconducting solenoid.

## Additional Information

**How to cite this article**: Nguyen, H. Q. *et al*. Driving a Superconductor to Insulator Transition with Random Gauge Fields. *Sci. Rep.* **6**, 38166; doi: 10.1038/srep38166 (2016).

**Publisher's note:** Springer Nature remains neutral with regard to jurisdictional claims in published maps and institutional affiliations.

## Supplementary Material

Supplementary Information
