# Supplementary material for: Driving a Superconductor to Insulator Transition with Random Gauge Fields
Source: Sci Rep. 2016 Nov 30;6:38166. doi: 10.1038/srep38166 (PMC5128869; doi:10.1038/srep38166)
Supplement: Supplementary Information [file srep38166-s1.pdf]

Supplementary Information for
Driving a Superconductor to Insulator Transition with Random Gauge Fields

H. Q. Nguyen,* S. M. Hollen,† and J. M. Valles Jr.
Department of Physics, Brown University, Providence, RI 02912

J. Shainline‡ and J. M. Xu
School of Engineering, Brown University, Providence, RI 02912

This supplemental information provides 1) a description of the magnetoresistance oscillations exhibited by NHC films and how they are influenced by flux disorder and 2) an estimate of magnetic field effects on J that could compete with the gauge field disorder influence that shows them to be of secondary importance at the low magnetic fields employed in the experiments.

Magnetoresistance Oscillations

Magnetoresistance oscillations are shown for two of the films in the supplemental figure. The minima in the oscillations occur at the commensurate fields. Notice that for the 20 kΩ film, the higher temperature red trace lies at or below the lower temperature blue trace for all of the minima. This behavior indicates that this film insulates at $\bar{f} = \bar{\phi}/\phi_0 = 1, 2, 3$. The more strongly coupled 19 kΩ film, on the other hand, insulates only for $\bar{\phi}/\bar{\phi}_0 = 2, 3$. The oscillation amplitude decreases with increasing field until it disappears at a critical field near $\bar{f} = 3$. As noted in the main text, this critical field value agrees with numerical simulations on square arrays[1] and hexagonal arrays[2] and experiments[3].

This agreement implies that other disorder effects, like a variation in J from link to link, exerts a relatively small influence on the low field magnetotransport and thus, the behavior at the low commensurate fields. One would expect that variations in J would cause the oscillations to decay more rapidly provided they were strong enough. This randomness in J could arise from variations in the local substrate surface. Such variations would cause the oscillations to wash out at a lower magnetic field. Indeed, the absence of structure in the magneto-resistance oscillations at $\bar{f} = 1/3$ suggests that there is some J_{ij} disorder in these films[4]. Nevertheless, these smaller variations in J_{ij} are expected to exert a relatively weak effect according to simulations[5]. Those simulations showed that the resistance as a function of temperature for arrays with no disorder and arrays with ±40% variation in J_{ij} at $\bar{\phi}/\bar{\phi}_0 = 0$ were very similar. Thus, it seems reasonable that the flux disorder effects dominate this other disorder effect at these low fields.

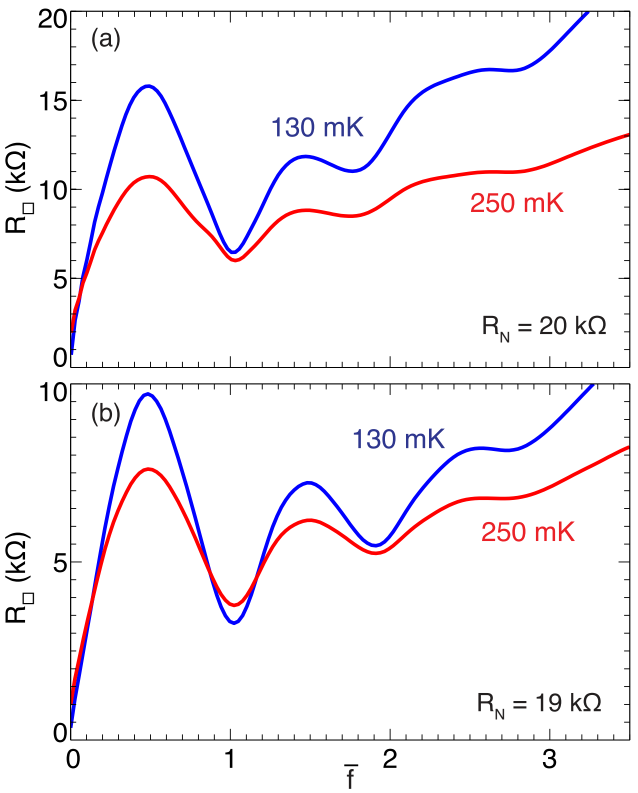

FIG. 1. Magnetoresistance oscillations of two films. a) R_N = 20 kΩ and b) R_N = 19 kΩ films shown in Fig. 1 of the main text. The oscillation minima correspond to the commensurate fields.

Magnetic Field Effects on J

A confounding magnetic field effect would be the reduction of J due to pair breaking in the nodes or links. Indeed, magnetic fields could reduce the order parameter amplitude in the nodes to reduce J to drive an SIT. This effect, however, only becomes appreciable at much higher fields that are comparable to the upper critical magnetic field, $\bar{\phi}/\phi_0 \approx 12$[6]. We consider this pairbreaking influence quantitatively in the main text and include it in the phase diagram figure.

Also, mesoscopic fluctuation effects in magnetic field might influence the J. However, in order for the magnetic field to exert a direct influence on the links, the

field must be high enough to fit a flux quantum in a link[7]. The geometry of the arrays dictates that $\bar{\phi}/\phi_0 \approx 6$ is required, which is higher than the field employed here. Thus, it seems reasonable that these effects are not apparent either.

---

* Currently at: Nano and Energy Center, Hanoi University of Science, Vietnam National University, Hanoi, Vietnam
† Currently at: Department of Physics, University of New Hampshire, Durham, NH 03824
‡ Currently at: National Institute of Standards and Technology, 325 Broadway, Boulder, Colorado, 80305, USA
[1] SP Benz, MG Forrester, M Tinkham, and CJ Lobb, "Positional disorder in superconducting wire networks and josephson junction arrays," Physical Review B **38**, 2869 (1988).
[2] Enzo Granato, "Magnetic flux disorder and superconductor-insulator transition in nanohole thin films," Phys. Rev. B **94**, 060504 (2016).
[3] MG Forrester, Hu Jong Lee, M Tinkham, and CJ Lobb, "Positional disorder in josephson-junction arrays: Experiments and simulations," Physical Review B **37**, 5966 (1988).
[4] Enzo Granato, "Superconductor-insulator transition of josephson-junction arrays on a honeycomb lattice in a magnetic field," The European Physical Journal B **89**, 1–8 (2016).
[5] Enzo Granato, "Resistive transition in frustrated josephson-junction arrays on a honeycomb lattice," Physical Review B **87**, 094517 (2013).
[6] HQ Nguyen, SM Hollen, MD Stewart Jr, J Shainline, Aijun Yin, JM Xu, and James M Valles Jr, "Observation of giant positive magnetoresistance in a cooper pair insulator," Physical review letters **103**, 157001 (2009).
[7] B.L. Altshuler and B.Z. Spivak, "Mesoscopic fluctuations in a superconductor-normal metal-superconductor junction," Soviet Physics JETP 65 (2), February 1987 **65**, 343–347 (1987).
